# Understanding the Dimensions of Caregiver-Perceived Seasonal Malaria Chemoprevention Delivery Quality in Nigeria: A Structural Equation Modeling Analysis of Door-to-Door Seasonal Malaria Chemoprevention

**DOI:** 10.4269/ajtmh.25-0661

**Published:** 2026-08-25

**Authors:** Tingkai Zhang, Sikai Huang, Taiwo Ibinaiye, Olusola Oresanya, Kevin Baker, Sol Richardson

**Affiliations:** ^1^ Vanke School of Public Health, Tsinghua University, Beijing, China;; ^2^ Malaria Consortium Nigeria, Abuja, Nigeria;; ^3^ Department of Global Public Health, Karolinska Institute, Stockholm, Sweden

## Abstract

Seasonal malaria chemoprevention (SMC) with sulfadoxine–pyrimethamine and amodiaquine (AQ) has been implemented in Africa since 2012 to prevent malaria among children ages 3 to 59 months old. As SMC expands to new regions, concerns are emerging about sustainability and quality of implementation. Successful SMC delivery depends on not only effective drug administration but also caregiver engagement, adherence to at-home AQ administration, retention of Child Record Cards, and overall malaria prevention practices. Although caregivers’ perceptions of delivery quality as well as knowledge and trust of SMC may play critical roles in shaping these behaviors, these associations remain underexplored. We conducted secondary analyses of SMC delivery quality and its associations with key SMC outcomes using two cross-sectional end-of-round SMC coverage surveys in 11 Nigerian states in 2021 and 2022 (*N* = 22,122). Exploratory factor analysis identified a single latent factor representing perceived SMC delivery quality based on six items reflecting distributor behavior and quality of communication. Logistic regression models revealed significant positive associations between higher index scores and days 2 and 3 AQ adherence as well as Child Record Card retention. Higher delivery quality was associated with reduced caregiver refusal of SMC. Structural equation modeling showed that perceived SMC delivery quality had significant direct effects on all three outcomes, with indirect effects significant only for days 2 and 3 AQ adherence. Findings highlight that improving and monitoring caregiver-perceived SMC delivery quality is essential to sustaining SMC effectiveness and suggest the need for strengthened distributor training and community engagement.

## INTRODUCTION

Seasonal malaria chemoprevention (SMC) is a highly effective strategy aimed at preventing malaria among children ages 3 to 59 months old in regions with high malaria transmission.^1^^–^^3^ This intervention typically involves the administration of sulfadoxine–pyrimethamine (SP) combined with amodiaquine (AQ) over annual rounds of four to five annual cycles during the high-transmission period where malaria transmission is seasonal. Seasonal malaria chemoprevention is crucial in reducing malaria incidence, particularly in sub-Saharan Africa, where malaria remains a significant public health challenge.^4^^–^^7^

Seasonal malaria chemoprevention is typically implemented through door-to-door delivery by trained SMC community distributors. During each visit, community distributors (defined as stipend-supported volunteers, the majority of whom are community health workers in Nigeria) administer one dose of SP and AQ on the first day (day 1), and they provide caregivers with instructions and doses for the subsequent days (day 2 and day 3). Caregivers are then responsible for administering the remaining AQ doses on day 2 and day 3. Seasonal malaria chemoprevention administration is expected to provide a period of protection against *Plasmodium falciparum* malaria over a 28-day period, which is referred to as an “SMC cycle.” Caregivers are also instructed to document the administration of each dose on SMC Child Record Cards, and they are instructed to retain these cards for monitoring purposes.^8^^,^^9^ In Nigeria, annual SMC rounds consist of four or five monthly cycles depending on the specific regions and duration of the transmission season. These rounds ensure continuous protection against malaria throughout the high-transmission period by repeating the SMC cycles monthly.^10^^,^^11^

Previous studies have primarily explored caregiver experiences, acceptability, and preferences regarding door-to-door SMC delivery using qualitative approaches.^12^ However, there is limited evidence on quantitatively measured caregiver-perceived delivery quality and its relationship with caregiver behaviors. Addressing these research gaps is essential for optimizing SMC delivery and maximizing its impact on malaria prevention.

This study aimed to examine how caregiver-perceived SMC delivery quality is associated with caregiver knowledge, attitudes, and SMC-related behaviors and outcomes in Nigeria. Objectives included 1) developing a comprehensive scale to assess caregiver-perceived SMC delivery quality with distributor interactions; 2) analyzing how caregiver-perceived SMC delivery quality is associated with key malaria prevention outcomes; and 3) exploring mediated pathways where caregiver-perceived SMC delivery quality influences their understanding and attitudes toward SMC, ultimately impacting malaria-related outcomes.

## MATERIALS AND METHODS

### Sampling and data collection.

This study is a secondary analysis of data from two cross-sectional end-of-round SMC coverage surveys conducted in Nigeria in 2021 and 2022 by external contractors not involved in SMC implementation.^13^ During sampling, survey clusters (communities) within each state were selected using probability proportional to population size, with 10 households sampled per cluster. Within each community, households were selected using simple random sampling, typically from local household lists. Within each household, data collectors compiled a roster of all children ages 3 to 119 months old. From this roster, children ages 3 to 59 months old were eligible, and one child was randomly selected using SurveyCTO. Detailed information on survey sampling and the protocol is available in the survey protocol and related publications.^10^^,^^14^

For data collection, primary caregivers of the selected children responded to the survey, which included questions about the child, the primary caregiver, and the household. The questionnaire also covered the child’s health status as well as the caregiver’s perceptions and knowledge related to SMC. This study included 8 states in 2021 (Bauchi, Borno, Kebbi, Kano, Kogi, Nasarawa, Plateau, and Sokoto) and 10 states in 2022 (Bauchi, Borno, Kebbi, Federal Capital Territory [FCT], Jigawa, Kogi, Nasarawa, Oyo, Plateau, and Sokoto). The geographic distribution of the survey areas across Nigeria is presented in Supplemental Figure 1.

### Conceptual framework.

We adopted a knowledge, attitudes, and practices (KAP) framework^15^^,^^16^ to explore the relationships between caregiver-perceived SMC delivery quality with distributor visits, their understanding and attitudes toward SMC, and subsequent malaria-related outcomes. Perceived delivery quality served as our core independent variable, whereas the KAP framework was used to structure the mediating variables (knowledge and attitudes) and outcome (practices). As this was a secondary analysis of existing survey data, the KAP framework did not guide the original questionnaire design. Instead, it guided our analytic approach and research question formulation as well as how we established the mediated variables and outcomes. Our hypothesis posited that caregiver-perceived SMC delivery quality not only is directly associated with improved adherence to SMC and malaria-related outcomes but also operates through indirect pathways mediated by caregivers’ knowledge and perceptions of SMC.

### Perceived SMC delivery quality.

Perceived SMC delivery quality with distributor visits was assessed based on caregivers’ agreement with six statements regarding SMC information and distributor interactions: “I believe the information given to me about SMC by the SMC distributors is sufficient,” “I believe the information given to me about SMC by the SMC distributors is easy to understand,” “I believe the information given to me about SMC by the SMC distributors is relevant to my family,” “I believe the information given to me about SMC by the SMC distributors is trustworthy,” “I believe the SMC distributor treated me (and/or my family) with respect during the last visit,” and “I believe the SMC distributor who visited our household during the last visit was adequately trained.” These items were originally adapted from the SMC Alliance monitoring and evaluation framework and the existing literature on SMC delivery quality.^17^^–^^19^

Caregivers responded using a five-point Likert scale (1 = strongly disagree to 5 = strongly agree). Scores were summed across the six items to generate a continuous perceived SMC delivery quality scale ranging from 6 to 30, with higher scores indicating more positive perceptions. Exploratory factor analysis with varimax rotation was used to assess the suitability of the perceived SMC delivery quality scale.

### Knowledge and perceptions.

Caregiver knowledge regarding SMC was assessed based on their responses to five binary questions: “Do you understand the purpose of SMC?,” “Do you know the age range of children eligible for SMC?,” “Do you know why only children aged under 5 years are eligible for SMC?,” “Do you recognize the importance of administering both day 2 and day 3 doses of AQ during SMC cycles?,” and “Do you know what to do in case of adverse reactions to SMC medicines?” Each question was answered with a “yes” or “no.” Caregiver knowledge was categorized into a binary variable: incomplete knowledge (zero to four correct answers) or full knowledge (five correct answers).

Caregiver attitudes toward SMC were evaluated using the following question: “Do you believe that SMC medicines are effective at protecting young children from malaria during the rainy season?” Caregivers responded with either “yes” or “no.”

### Seasonal malaria chemoprevention and malaria-related outcomes.

We examined several key outcome variables related to SMC and its impact on malaria-related outcomes among caregivers. These included adherence to the administration of both day 2 and day 3 AQ by caregivers in the last SMC cycle. We also considered instances where caregivers refused SMC for their children and retention of the SMC Child Record Cards by caregivers. Each of these outcome variables was coded as binary. These outcomes collectively provide insights into the effectiveness of SMC implementation and its influence on malaria prevention practices among caregivers of SMC-eligible children in Nigeria.

### Covariates.

Covariates, which are hypothesized to be potential confounders for the relationship between perceived SMC delivery quality and outcomes, encompassed child and caregiver demographics, including children’s sex and age categorized into 1-year intervals and caregiver’s sex, age categorized into 10-year groups, partnership status (married/partnered or unpartnered), and highest educational attainment levels (no education, informal education, primary school, secondary school, or higher education). Analyses used state-level survey weights based on the size of state-level populations to ensure representativeness of the states included in the analysis.

## STATISTICAL ANALYSES

Caregivers’ responses to perceived SMC delivery quality-related statements were subjected to exploratory factor analysis using varimax rotation. This approach was chosen to examine the underlying structure of the perceived SMC delivery quality scale derived from six Likert-scale items. The factor analysis aimed to validate the scale and identify any underlying dimensions that contribute to caregiver-perceived SMC delivery quality with SMC distributor visits.

Logistic regression models were used to assess the associations between the perceived SMC delivery quality scale and the SMC- and malaria-related outcome variables among caregivers in Nigeria. We tested the associations between the scale and each of the outcomes after adjustment for covariates; analyses used state-level survey weights.

To further explore the complex pathways within the KAP framework, structural equation modeling (SEM) was used. Because our analysis was hypothesis driven, we specified a single theoretically derived model rather than comparing alternative models. Structural equation modeling aimed to explore direct and mediated effects involving caregiver-perceived SMC delivery quality, knowledge, and perceptions related to SMC as well as various malaria-related outcomes. Caregiver’s knowledge about SMC and their perceptions of SMC were considered as potential mediators in the SEM analysis. We estimated both unadjusted and adjusted SEM, with covariates included in the latter to control for potential confounding factors. Model fit was assessed using the comparative fit index (CFI) and the root mean square error of approximation (RMSEA). All statistical analyses used state-level survey weights.

## RESULTS

### Caregiver-perceived SMC delivery quality scale.

**Figure 1.**
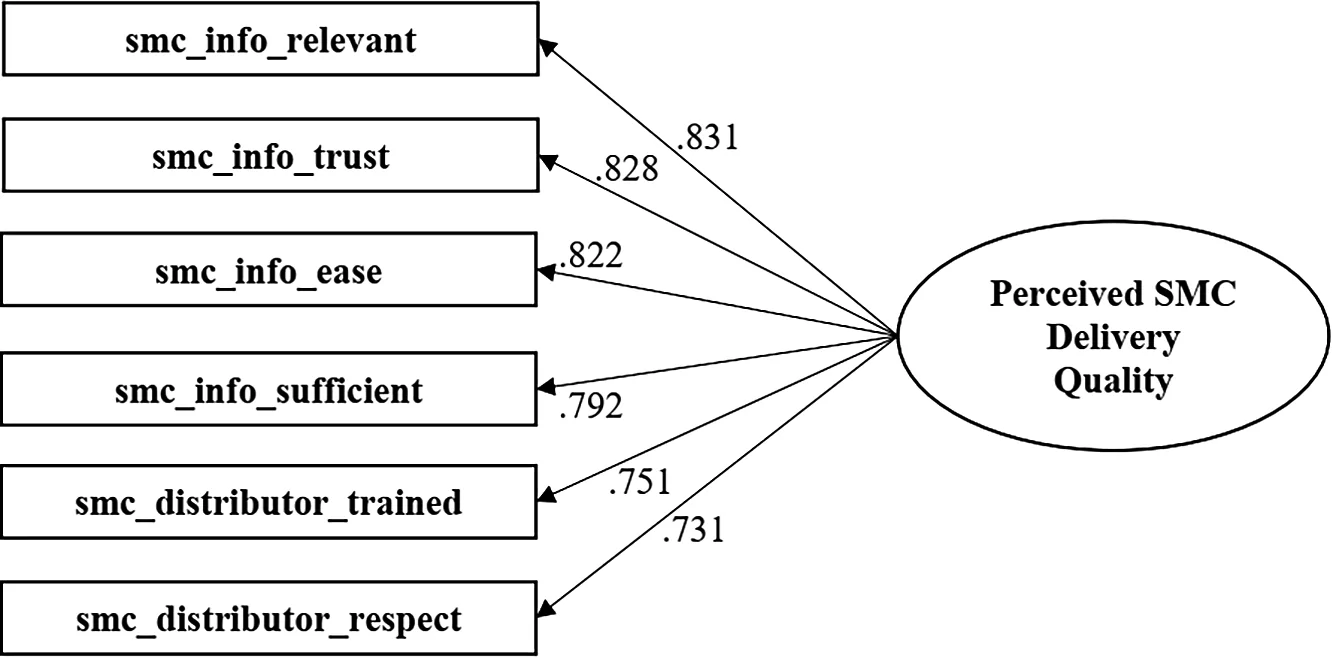
Exploratory factor analysis loading diagram. SMC = seasonal malaria chemoprevention.

Based on exploratory factor analysis (Figure 1), a one-factor solution encompassing all six perceived SMC delivery quality items demonstrated the most appropriate scale for Nigerian respondents based on assessment of eigenvalues. This analysis confirmed high internal consistency with a Cronbach alpha of 0.91, indicating strong reliability of the scale. The factor loadings for the perceived SMC delivery quality items are as follows: smc_info_relevant (0.831), smc_info_trust (0.828), smc_info_ease (0.822), smc_info_sufficient (0.792), smc_distributor_trained (0.751), and smc_distributor_respect (0.731). This indicates that all items contribute significantly to the overall caregiver-perceived SMC delivery quality construct.

In total, 59.9% of caregivers scored 25 or higher on the perceived SMC delivery quality scale from 5 to 30 points, reflecting generally high levels of perceived SMC delivery quality with the door-to-door delivery of SMC in Nigeria. The distribution of scores deviated from normality, necessitating appropriate statistical adjustments in subsequent analyses.

### Participant characteristics.

Table 1 presents the demographic characteristics of the 22,212 participating caregivers and their children. The majority were female (83.1%), ages 20 to 39 years old (79.8%), and married or partnered (95.2%). Approximately half of caregivers (47.6%) had no formal or only informal education. Child age was distributed across the 3- to 59-months-old range, with slightly more children in the 48- to 59-months-old group (27.1%), and child sex was balanced (48.6% female and 51.4% male).

**Table 1:** Demographic characteristics of participating caregivers with children ages 3–59 months old in Nigeria (*N* = 22,212)

Characteristics	Percentage
Children	
Age (months)	
3–11	6.72
12–23	16.77
24–35	23.96
36–47	25.43
48–59	27.12
Sex	
Female	48.60
Male	51.40
Children’s caregiver (years)	
Age	
Younger than 20	5.13
20–29	42.93
30–39	36.88
40–49	11.29
50 or older	3.77
Sex	
Female	83.10
Male	16.90
Education level	
No education	23.94
Informal education	23.61
Primary school	16.45
Secondary school	28.03
Higher education	7.97
Partnership	
Married or partnered	95.23
Unpartnered	4.77
SMC outcomes	
Day 2 and day 3 AQ adherence	
Yes	97.09
No	1.52
Missing	1.39
Retention of SMC child record card	
Yes	89.72
No	10.28
Refusal of day 1 SP + AQ	
Yes	0.07
No	98.61
Missing	1.32
Knowledge and perceptions	
Caregivers’ full knowledge of SMC	
Yes	61.22
No	38.78
Caregivers’ perceived effectiveness of SMC	
Yes	85.01
No	14.99

AQ = amodiaquine; SMC = seasonal malaria chemoprevention; SP = sulfadoxine–pyrimethamine.

In terms of SMC outcomes, adherence to day 2 and day 3 AQ administration was high (97.1%) as was retention of SMC Child Record Cards (89.7%). Refusal of day 1 SP and AQ was very low (0.1%). Regarding knowledge and attitudes, 61.2% of caregivers had full knowledge of SMC, and 85.0% believed that SMC is effective at preventing malaria.

### Association between perceived SMC delivery quality and outcomes.

Table 2 presents the logistic regression results for the association between caregiver-perceived SMC delivery quality and key SMC-related outcomes. Significant associations were observed between caregiver-perceived SMC delivery quality scores and key SMC-related outcomes. There was a positive association between each point increase in perceived SMC delivery quality scores and adherence to day 2 and day 3 AQ administration (odds ratio [OR]: 1.208, 95% CI: 1.160–1.258, *P* <0.001). Caregivers with higher perceived SMC delivery quality scores per point were also more likely to retain SMC Child Record Cards (OR: 1.108, 95% CI: 1.090–1.127, *P* <0.001).

**Table 2:** Logistic regression results for the association between perceived seasonal malaria chemoprevention delivery quality and outcomes

Outcomes	OR (95% CI)	*P*-Value
Day 2 and day 3 AQ adherence	1.208 (1.160–1.258)	<0.001
Retention of SMC Child Record Cards	1.108 (1.090–1.127)	<0.001
Refusal of day 1 SP + AQ	0.706 (0.616–0.810)	<0.001

AQ = amodiaquine; OR = odds ratio; SMC = seasonal malaria chemoprevention; SP = sulfadoxine–pyrimethamine. All regression models are adjusted for child’s age, child’s sex, caregiver’s age, caregiver’s gender, caregiver’s level of education, and caregiver’s partnership status.

Conversely, higher perceived SMC delivery quality scores per point were negatively associated with caregiver refusal of day 1 SP and AQ (OR: 0.706, 95% CI: 0.616–0.810, *P* <0.001).

### Assessment of mediated associations.

Table 3 presents the logistic regression results for the associations between caregiver-perceived SMC delivery quality and knowledge and caregiver perceptions of SMC. It showed a significant positive association between caregiver-perceived SMC delivery quality and full knowledge of SMC (OR: 1.018, 95% CI: 1.005–1.030, *P* = 0.005). There was strong positive association between caregiver-perceived SMC delivery quality and perceived effectiveness of SMC (OR: 1.211, 95% CI: 1.171–1.253, *P* <0.001), suggesting that caregivers who report higher perceived SMC delivery quality are significantly more likely to perceive SMC as effective.

**Table 3:** Logistic regression results for the associations between perceived seasonal malaria chemoprevention delivery quality, knowledge, and perceptions of caregivers toward seasonal malaria chemoprevention in Nigeria

Mediator Outcomes	OR (95% CI)	*P*-Value
Full knowledge	1.018 (1.005–1.030)	0.005
Perceived effectiveness	1.211 (1.171–1.253)	<0.001

OR = odds ratio. All regression models are adjusted for child’s age, child’s sex, caregiver’s age, caregiver’s gender, caregiver’s level of education, and caregiver’s partnership status.

**Figure 2.**
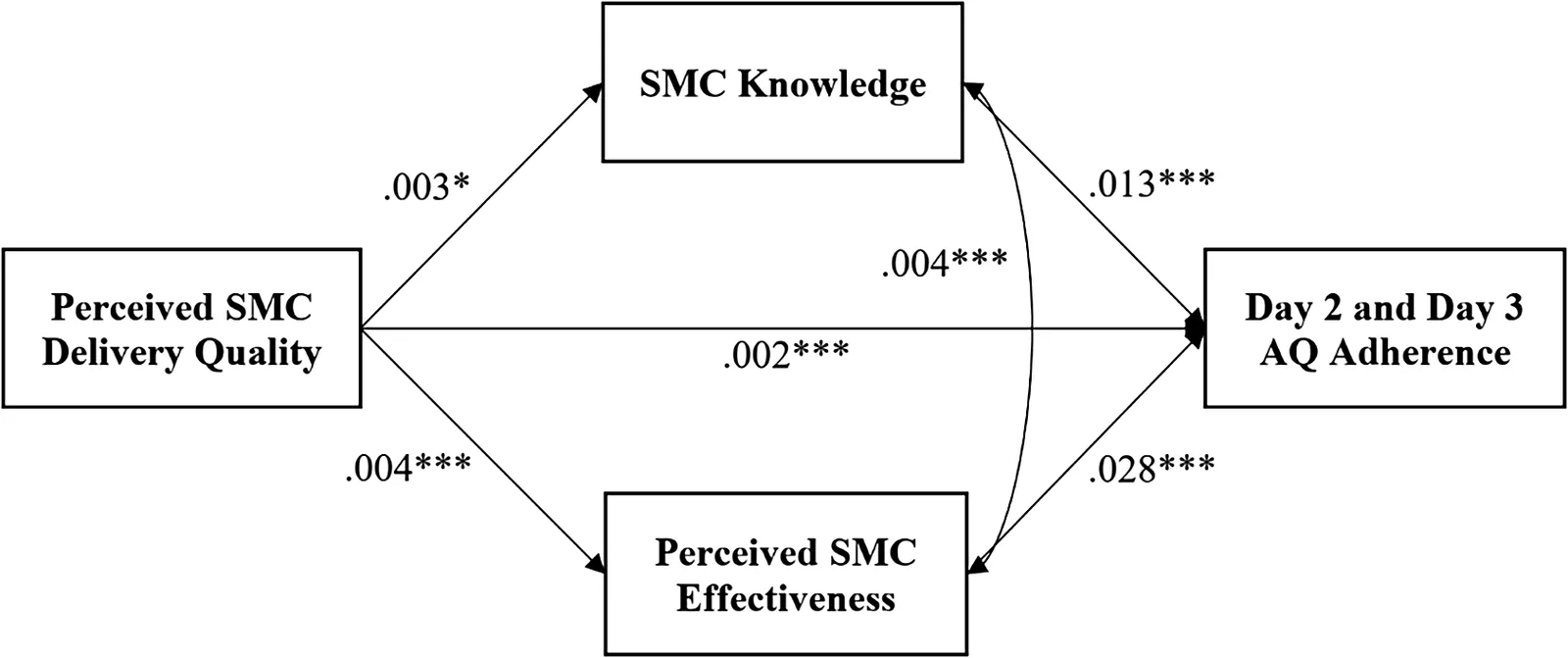
Adjusted structural equation model showing associations between caregiver-perceived seasonal malaria chemoprevention (SMC) delivery quality, caregiver knowledge and perceptions, and adherence to day 2 and day 3 amodiaquine (AQ) administration. The model is adjusted for child age and sex and caregiver age, sex, education, and partnership status. *P <0.05; ***P <0.001.

The SEM analysis illustrated in Figure 2 is as an example representing the association between caregiver-perceived SMC delivery quality, caregiver adherence to AQ administration, and the mediation of caregiver’s knowledge and perceptions of SMC. The model demonstrated good fit, which are indicated by a CFI of 1.000 and an RMSEA of 0.000. There was a positive direct effect of caregiver-perceived SMC delivery quality on adherence to administering day 2 and day 3 doses of AQ (β = 0.0023, 95% CI: 0.0017–0.0029, *P* <0.001). The indirect effect was 0.0002 (95% CI: 0.0001–0.0002, *P* <0.001), and the total effect was 0.0024 (95% CI: 0.0018–0.0031, *P* <0.001). Unadjusted results for day 2 and day 3 AQ administration adherence are presented in Supplemental Figure 2.

Applying the SEM model to all three outcomes, the direct effects of perceived SMC delivery quality on adherence, card retention, and refusal were all statistically significant (*P* <0.001). The indirect effect was significant for adherence (*P* <0.001) but not for retention (*P* = 0.273) or refusal (*P* = 0.097). Full adjusted SEM results are presented in Supplemental Table 1.

## DISCUSSION

Our study identified three key findings regarding caregiver-perceived SMC delivery quality with SMC delivery in Nigeria. First, caregiver-perceived SMC delivery quality was effectively measured using a one-factor, six-item scale demonstrating high internal consistency. Second, we observed significant associations between caregiver-perceived SMC delivery quality and a range of SMC outcomes. Third, the effects of perceived SMC delivery quality on SMC outcomes were primarily direct, with significant direct effects on all three outcomes. Indirect effects through knowledge and attitudes were only significant for day 2 and day 3 AQ adherence.

We found that a six-item, single-factor scale reliably measured caregiver-perceived SMC delivery quality, demonstrating high internal consistency and establishing its validity in the Nigerian context. The single-factor structure suggests that caregivers perceive distributor behavior and communication quality as a unified construct rather than distinct dimensions. The high internal consistency indicates that the six items reliably capture this construct, supporting its use as a composite index in future SMC program monitoring.

Caregiver-perceived SMC delivery quality was positively associated with day 2 and day 3 AQ adherence as well as retention of SMC Child Record Cards. This is consistent with evidence from other preventive and treatment contexts, including HIV care, where perceived quality of service delivery has been linked to improved adherence to antiretroviral therapy.^20^^–^^22^ Similar mechanisms may underlie these associations, where higher perceived delivery quality enhances trust and satisfaction, directly leading to better adherence and retention behaviors. Notably, full adherence to the SMC regimen is essential for achieving optimal chemopreventive effectiveness,^23^^,^^24^ and nonadherence may lead to accelerated development of AQ resistance in populations targeted for SMC.^25^^–^^27^ Given that adherence directly determines individual protection and influences population-level drug exposure, factors associated with improved adherence are of substantial public health importance. In this context, our finding that caregiver-perceived delivery quality is associated with better adherence suggests that service delivery experience may play a meaningful role in optimizing the effectiveness of SMC programs. However, other studies pointed to the predominant role of personal motivation and health concerns in determining adherence to interventions involving adherence to medicine courses as opposed to quality of care or influence from health care workers.^28^

Higher perceived SMC delivery quality scores per point were negatively associated with caregiver refusal of day 1 SP and AQ. This suggests that higher perceived quality reduces the likelihood of refusal, likely because of increased trust and confidence in the program, similar to the positive associations observed with adherence and SMC Child Record Card retention. Interestingly, perceived SMC effectiveness was not significantly associated with refusal, possibly because refusal is a more immediate, trust-based decision made at the doorstep rather than one driven by abstract beliefs about malaria prevention.

Structural equation modeling further revealed that the effects of perceived SMC delivery quality on SMC outcomes were predominantly direct. Although knowledge and attitudes partially mediated the association with day 2 and day 3 AQ adherence, they did not significantly mediate the effects on card retention of SMC Child Record Cards or refusal. This suggests that perceived delivery quality influences caregiver behaviors primarily through direct pathways, such as immediate trust, satisfaction with the distributor interaction, or perceived convenience, rather than through caregivers’ knowledge of SMC or their beliefs about its effectiveness. From a program perspective, this indicates that improving the quality of distributor visits can directly and immediately impact caregiver behaviors.

The findings underscore the importance of enhancing distributor training to ensure consistent and high-quality SMC delivery that meets caregiver expectations. Additionally, expanding coverage of sensitization activities and tailoring information channels and content to fit the needs of diverse audiences are crucial steps. Particularly, leveraging face-to-face engagement by community leaders can effectively reach socioeconomically disadvantaged groups, enhancing program accessibility and effectiveness.

Despite the perceived SMC delivery quality directly influencing SMC adherence, previous literature has found that health message dissemination can also improve outcome adherence.^29^^,^^30^ Therefore, SMC campaigns present an opportunity beyond the delivery of malaria-preventing medicines; they can serve as platforms for disseminating broader health messages to both enhance impact of SMC in malaria prevention and achieve other secondary impacts.

To sustain caregiver engagement and support, we recommend integrating caregiver-perceived SMC delivery quality assessments into routine end-of-cycle surveys. This proactive approach allows for prompt identification of communities with lower perceived SMC delivery quality levels, enabling targeted interventions to maintain trust, enhance engagement, ensure sustainability of the intervention, and optimize outcomes. Also, improving perceived delivery quality is a modifiable program factor that does not require additional intervention inputs but relies on strengthening existing delivery practices through cascade training, making it a cost-effective strategy to enhance SMC impact.

### Strengths.

The study leveraged routine end-of-round coverage survey data with a wide range of relevant variables, which provided a cost-effective means to test associations between caregiver-perceived delivery quality and multiple outcomes while adjusting for multiple covariates. This approach facilitated comprehensive exploration of caregiver-perceived SMC delivery quality with SMC distributor visits and its impact on various outcomes.

### Limitations.

Several limitations, however, must be considered. First, the study’s cross-sectional design precludes establishing causal relationships between caregiver-perceived SMC delivery quality with SMC distributor visits and SMC-related and malaria outcomes. Second, reliance on self-reported data from caregivers introduces potential biases, such as recall and social desirability biases, which may influence reliability of responses. Third, the study lacked data on long-term malaria outcomes, limiting the ability to assess associations between SMC knowledge, perceptions, perceived SMC delivery quality, and sustained malaria prevention effectiveness. Moreover, some surveyed areas in Nigeria had received mass SMC delivery since 2013, potentially influencing caregiver knowledge and perceptions as these may have been established in previous years.

## CONCLUSION

Our study found significant associations between perceived delivery quality and key SMC outcomes, with effects being predominantly direct. These associations highlight the critical role of perceived SMC delivery quality in promoting and ensuring the effectiveness of SMC. To optimize SMC delivery and ensure sustainability, it is crucial to improve distributor training, expand sensitization activities, and leverage community engagement. Integrating quality assessments into routine surveys will sustain caregiver trust and support, ensuring better health outcomes and broader health promotion.

## Supplemental Materials

10.4269/ajtmh.25-0661 Supplemental Materials
