# Supplementary material for: Understanding the Dimensions of Caregiver-Perceived Seasonal Malaria Chemoprevention Delivery Quality in Nigeria: A Structural Equation Modeling Analysis of Door-to-Door Seasonal Malaria Chemoprevention
Source: Am J Trop Med Hyg. 2026 Aug 25;115(4):778–84. doi: 10.4269/ajtmh.25-0661 (PMC13527193; doi:10.4269/ajtmh.25-0661)
Supplement: Supplementary file 1 [file tpmd250661.SD1.pdf]

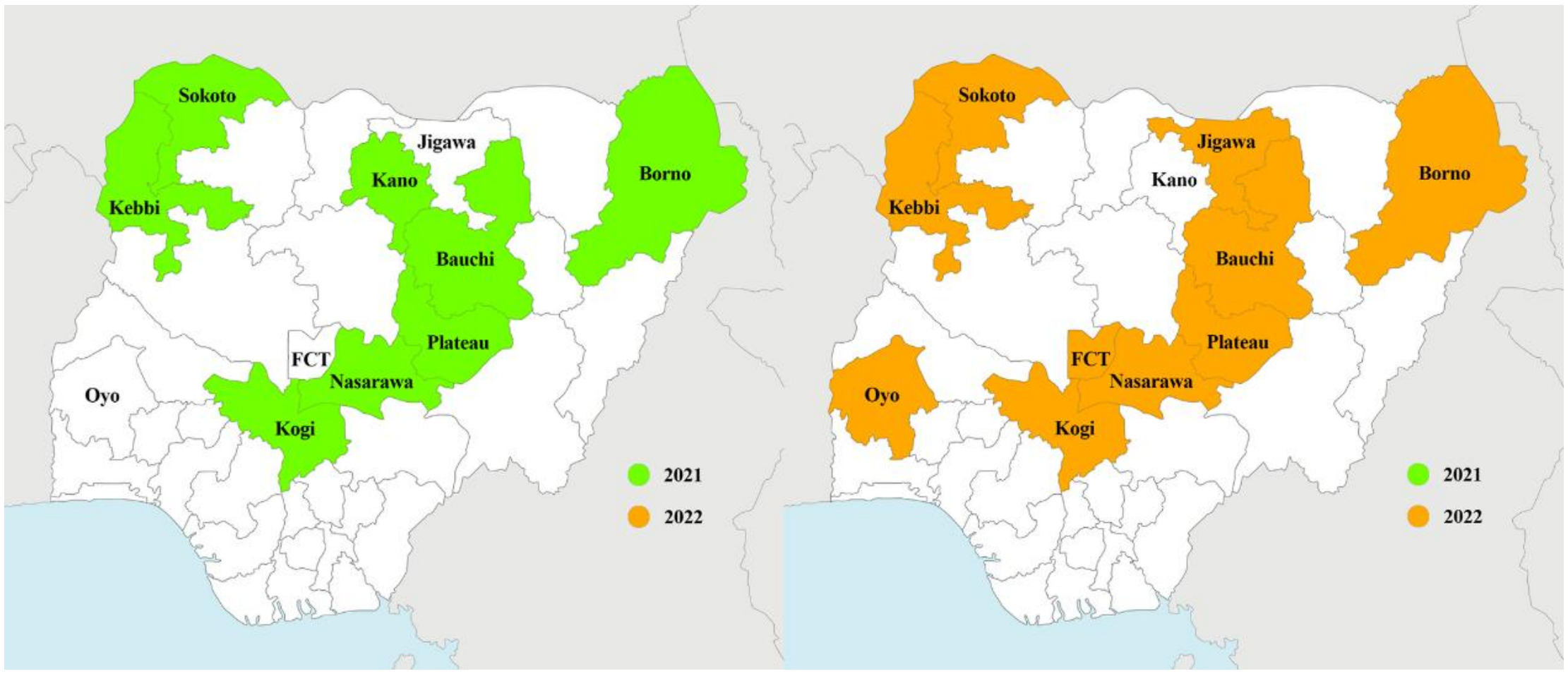

Supplemental Figure 1. Geographic coverage of the SMC survey conducted in Nigeria, 2021-2022.

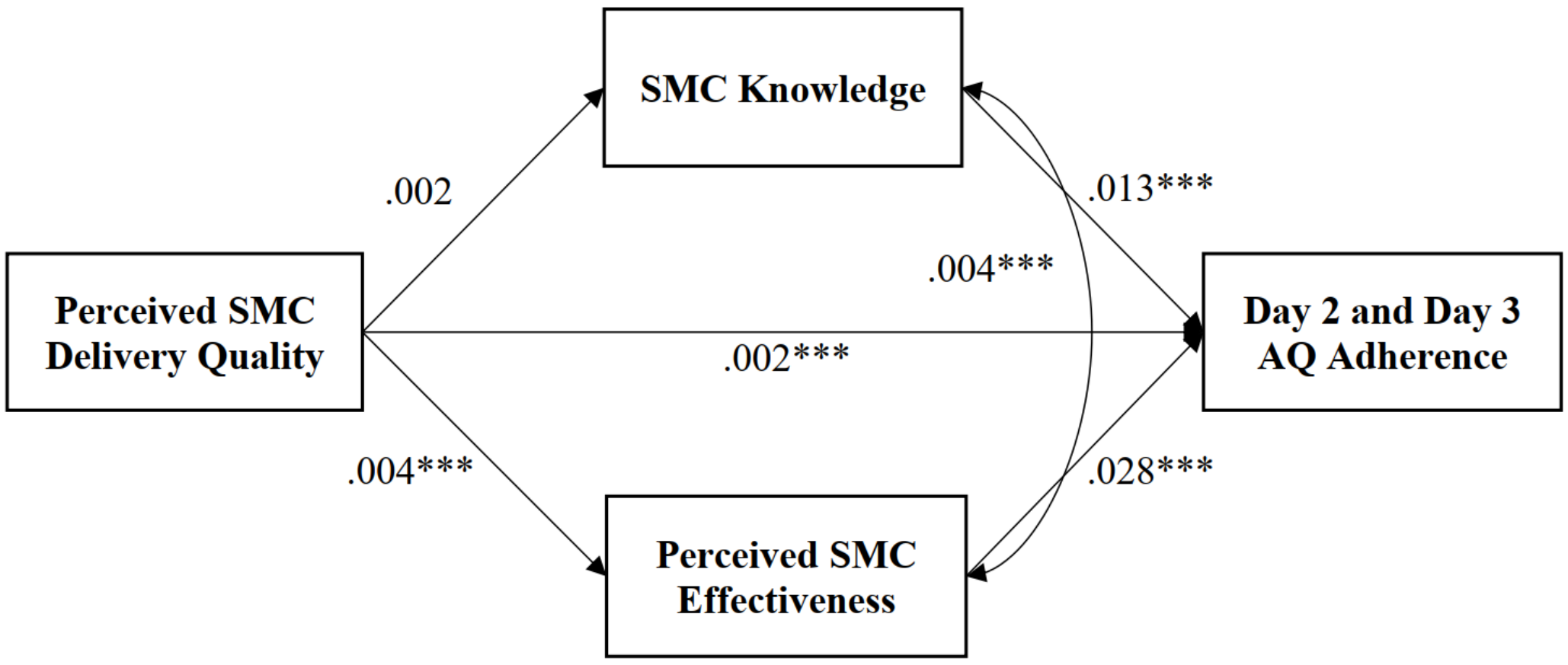

Supplemental Figure 2. Unadjusted structural equation model showing associations between caregiver-perceived SMC delivery quality, caregiver knowledge and perceptions, and adherence to day 2 and day 3 AQ administration.

*p<0.05, **p<0.01, ***p<0.001.

Supplemental Table 1

SEM results for the mediation of knowledge and attitudes on the association between perceived SMC delivery quality and three SMC outcomes

Outcome / Pathway	Coef. (95% CI)	*P*
**Day 2 & Day 3 AQ Adherence**		
X → K	0.0028 (0.0003 – 0.0052)	0.028
X → A	0.0044 (0.0035 – 0.0053)	0.000
K → P_1	0.0134 (0.0098 – 0.0170)	0.000
A → P_1	0.0279 (0.0177 – 0.0380)	0.000
Direct effect: X → P_1	0.0023 (0.0017 – 0.0029)	0.000
Indirect effect	0.0002 (0.0001 – 0.0002)	0.000
Total effect	0.0024 (0.0018 – 0.0031)	0.000
**Retention of SMC Child Record Cards**		
X → K	0.0034 (0.0009 – 0.0058)	0.007
X → A	0.0049 (0.0041 – 0.0058)	0.000
K → P_2	0.0956 (0.0866 – 0.1045)	0.000
A → P_2	-0.0350 (-0.0604 – -0.0097)	0.007
Direct effect: X → P_2	0.0086 (0.0070 – 0.0101)	0.000
Indirect effect	0.0001 (-0.0001 – 0.0004)	0.273
Total effect	0.0087 (0.0071 – 0.0103)	0.000
**Refusal of Day 1 SP+AQ**		
X → K	0.0029 (0.0004 – 0.0053)	0.022
X → A	0.0045 (0.0036 – 0.0053)	0.000
K → P_3	-0.0009 (-0.0016 – -0.0002)	0.010
A → P_3	-0.0012 (-0.0031 – 0.0008)	0.245
Direct effect: X → P_3	-0.0003 (-0.0004 – -0.0001)	0.000
Indirect effect	-0.0000 (-0.0000 – 0.0000)	0.097
Total effect	-0.0003 (-0.0004 – -0.0001)	0.000

X=Perceived SMC delivery quality; K=Full knowledge of SMC; A=Perceived SMC effectiveness; P_1=Adherence; P_2=Retention; P_3=Refusal. All models adjusted for covariates. Coefficients and 95% CIs rounded to four decimals; p-values rounded to three decimals; 0.000 indicates $p<0.001$. Indirect effects are the sum of pathways via K and A.
